# From injection to inhalation: A STING‐activating mucosal adjuvant reprograms vaccination

**DOI:** 10.1002/ctm2.70798

**Published:** 2026-08-25

**Authors:** Weijie Wang, Muzhuo Wang, Lu Lu, Shibo Jiang, Zezhong Liu

**Affiliations:** ^1^ Department of Pharmacology and the Key Laboratory of Smart Drug Delivery Ministry of Education, School of Pharmacy Key Laboratory of Medical Molecular Virology (MOE/NHC/CAMS) Shanghai Institute of Infectious Disease and Biosecurity School of Basic Medical Sciences Shanghai Fifth People's Hospital Fudan University Shanghai China

1

The respiratory mucosa of the nasal cavity and conducting airways is a major portal of entry for respiratory viruses and an important site for vaccine‐induced protection. Local immunity acts at the earliest stage of infection, when viral replication remains largely confined to the respiratory epithelium.^1^ Secretory immunoglobulin A (sIgA) is a key component of mucosal adaptive immunity and can block viral attachment to host receptors and limit entry into epithelial cells. Tissue‐resident memory T (T_RM_) cells can respond rapidly within the respiratory tract and help eliminate infected cells without relying on the recruitment of circulating lymphocytes. These responses are difficult to establish after intramuscular vaccination.^2^, ^3^ Intramuscular vaccines induce strong systemic antibody and T‐cell responses and reduce the risk of severe respiratory infection. However, their protection in the upper respiratory tract is often weaker and less durable. This limitation has renewed interest in mucosal vaccination.^4^, ^5^

Effective mucosal vaccines remain difficult to develop. Although several mucosal COVID‐19 vaccines based on adenoviral or influenza virus vectors have been authorized in some countries for aerosol inhalation or intranasal administration, approved protein‐subunit mucosal vaccines remain rare. Respiratory mucosal subunit vaccines face three main challenges. First, protein antigens must diffuse through the mucus layer and then be transported across, or sampled at, the epithelial barrier to reach antigen‐presenting cells (APCs) in the underlying lamina propria. During this process, they must also resist proteolytic degradation and rapid mucociliary clearance. Second, soluble protein antigens are often weakly immunogenic at mucosal surfaces and, without sufficient innate immune stimulation, may induce weak responses or mucosal tolerance. Third, approved adjuvanted protein‐based mucosal vaccines remain rare and no broadly established adjuvant platform is currently available for intranasal protein‐subunit vaccination (Figure 1).^6^, ^7^ An effective mucosal adjuvant must therefore promote rapid antigen uptake and presentation while limiting local inflammation and systemic exposure. Several established adjuvants have been tested through the nasal route. MF59 enhanced mucosal antibody responses in animal studies, while CpG oligodeoxynucleotides, such as CpG 1018, promoted both mucosal and systemic immunity. Neither MF59 nor CpG 1018 is approved for intranasal use. MF59 is licensed in injectable influenza vaccines and CpG 1018 is used in an injectable hepatitis B vaccine. Their safety and activity after injection cannot be directly extended to the respiratory tract.^8^, ^9^ Bacterial toxins and their derivatives, including cholera toxin‐based adjuvants, have shown stronger mucosal activity, but safety remains a major concern.^7^, ^10^ Therefore, developing safe and effective mucosal adjuvants with minimal systemic inflammatory effects remains a major challenge.

**FIGURE 1:**
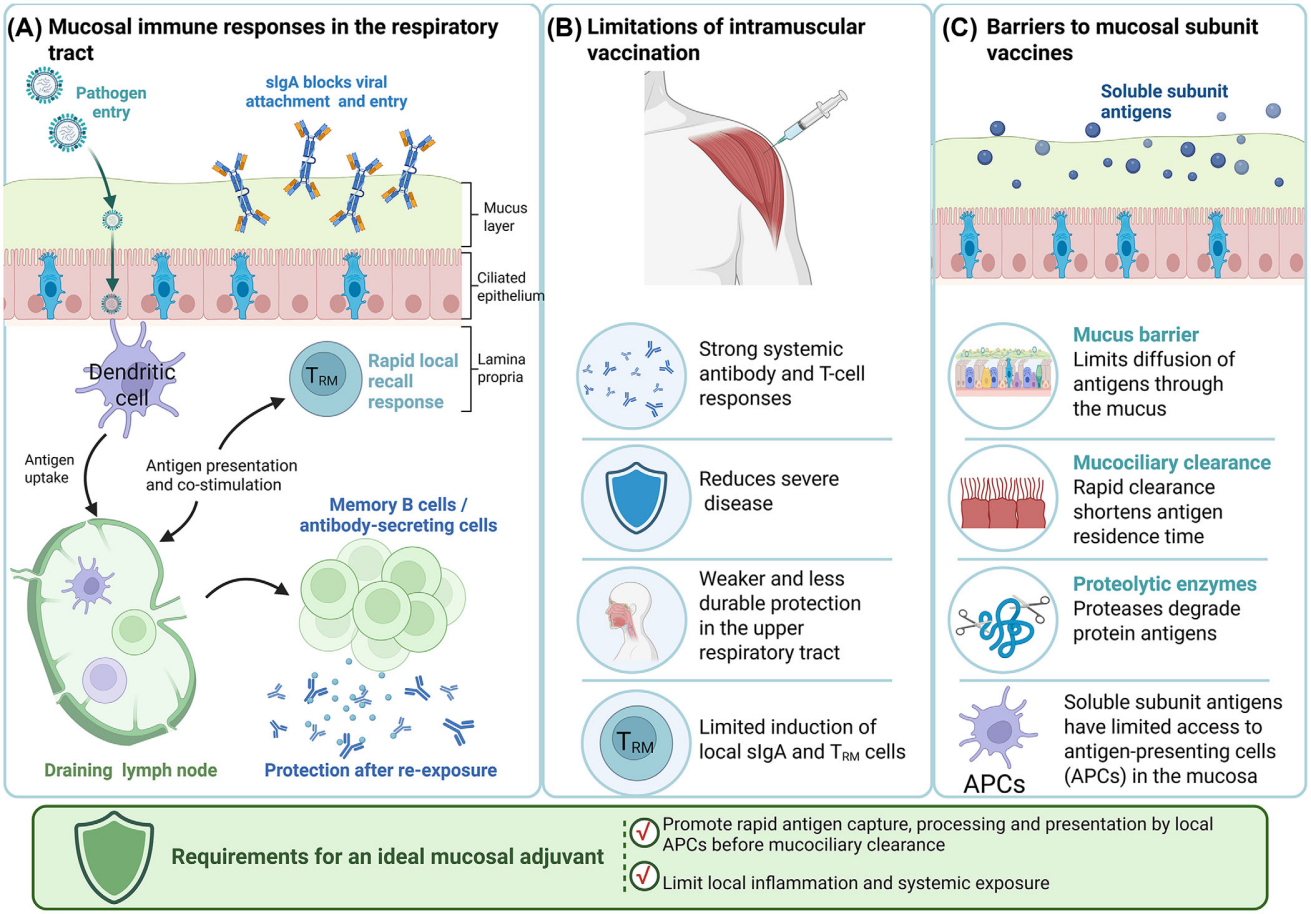
Mucosal immunity in the respiratory tract and major barriers to effective mucosal vaccination. (A) sIgA limits viral attachment and entry, while T_RM_ cells support rapid local responses in the respiratory tract. Memory B cells and antibody‐secreting cells contribute to protection after re‐exposure. (B) Intramuscular vaccination induces strong systemic antibody and T‐cell responses but generally provides weaker and less durable immunity in the upper respiratory tract. (C) Intranasally delivered subunit antigens encounter several physical and biological barriers, including restricted diffusion through mucus, rapid mucociliary clearance, degradation by proteolytic enzymes and inefficient access to local antigen‐presenting cells. An effective mucosal adjuvant should therefore promote rapid antigen capture, processing and presentation while limiting local inflammation and systemic exposure. sIgA, secretory immunoglobulin A; T_RM_, tissue‐resident memory T.

To address these challenges, we first identified CF501, a non‐nucleotide small‐molecule STING agonist with potent adjuvant activity. CF501 enhanced immune responses to poorly immunogenic conserved receptor‐binding domain (RBD) epitopes and induced broad neutralizing antibodies. Even with an ancestral‐strain RBD antigen, CF501‐adjuvanted vaccination elicited potent neutralization of immune‐evasive variants, including SARS‐CoV‐2 omicron XBB recombinant lineage (XBB).^11^, ^12^, ^13^, ^14^ Next, to adapt CF501 for respiratory mucosal vaccination, we formulated it with poly(2‐ethyl‐2‐oxazoline) into NanoCF501 particles of approximately 18 nm, with the aim of balancing respiratory compatibility, immunogenicity and safety. This resulting mucosal adjuvant achieved five key advances.^15^ First, NanoCF501 remained largely confined to the respiratory tract and limited systemic exposure, which may reduce the risk of systemic inflammation. Second, its small and uniform particle size was compatible with passage through respiratory mucus. After intranasal administration, NanoCF501 promoted dendritic‐cell (DC) trafficking to draining lymph nodes and enhanced DC maturation *in* *vivo*. Third, SSM2 was designed as a multivalent fusion protein in which tandemly linked receptor‐binding domains from SARS‐CoV‐2, SARS‐CoV and MERS‐CoV were fused to a human IgG1 Fc domain. Together, NanoCF501 and SSM2 induced broad mucosal and systemic immunity against β‐coronaviruses. The response included robust airway sIgA responses, respiratory T_RM_ cells and potent cross‐neutralizing antibodies, as well as protection against matched and heterologous β‐coronavirus challenges. Fourth, these immune responses were durable. Cross‐neutralizing activity persisted for at least 12 months and was accompanied by bonemarrow long‐lived plasma cells (LLPCs), splenic SARS‐CoV‐2 RBD‐specific memory B cells and persistent respiratory T_RM_ populations in mice. Fifth, the platform showed early translational potential. In rhesus macaques, NanoCF501/SSM2 induced mucosal and systemic antibodies, neutralizing activity and sustained RBD‐specific T‐cell responses. In mice, NanoCF501 also enhanced mucosal and systemic antibody responses to a licensed quadrivalent influenza subunit vaccine administered intranasally. More recently, we found that intranasal immunization combining a stimulator of interferon genes (STING) agonist with a nanoparticle‐displayed multivalent antigen markedly increased both the magnitude and breadth of the elicited immune responses in mice.^16^ Together, these findings suggest that integrating potent mucosal immune stimulation with rational multivalent antigen display may represent a broadly applicable strategy for developing respiratory mucosal vaccines with enhanced potency and breadth (Figure 2).

**FIGURE 2:**
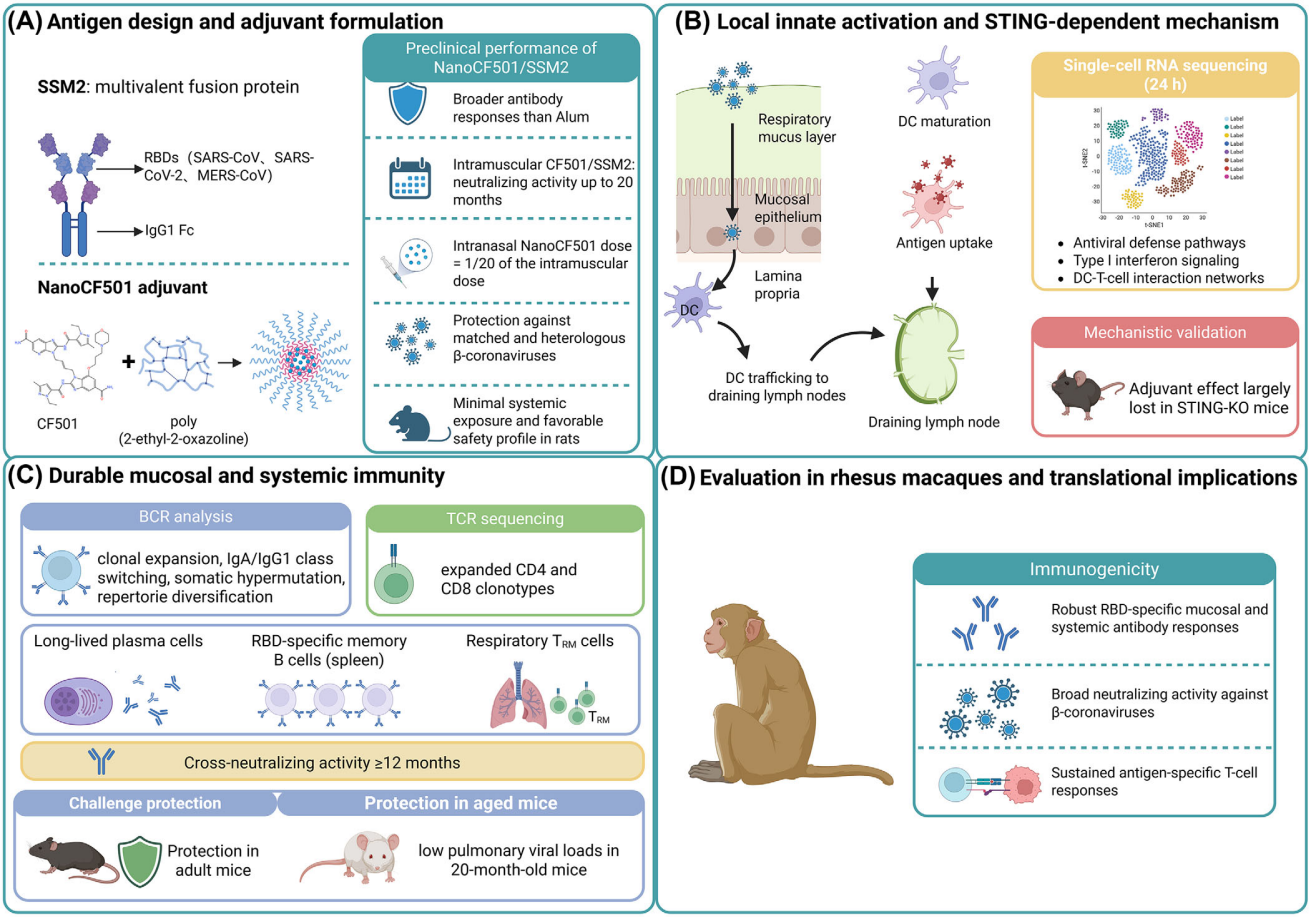
Design, mechanism and preclinical activity of the NanoCF501/SSM2 mucosal vaccine. (A) SSM2 comprises tandemly linked receptor‐binding domains from SARS‐CoV‐2, SARS‐CoV and MERS‐CoV fused to a human IgG1 Fc domain. CF501 was formulated with poly(2‐ethyl‐2‐oxazoline) to form NanoCF501 particles of approximately 18 nm. Intranasal NanoCF501/SSM2 induced broad mucosal and systemic immunity and protected mice against matched and heterologous β‐coronaviruses. At 1/20 of the intramuscular NanoCF501 dose, intranasal vaccination produced comparable systemic neutralizing antibody titres. Intranasal dosing in rats resulted in minimal systemic CF501 exposure. (B) NanoCF501 was compatible with passage through respiratory mucus, promoted dendritic‐cell trafficking and maturation in vivo and enhanced model‐antigen uptake in bone‐marrow‐derived dendritic cells. It induced transient local innate immune activation, and its adjuvant effect was largely lost in global STING‐knockout mice. (C) B‐cell receptor analysis showed greater clonal expansion, IgA/IgG1 class switching, somatic hypermutation and repertoire diversification, while T‐cell receptor sequencing showed greater CD4^+^ and CD8^+^ T‐cell clonotype expansion. Cross‐neutralizing activity persisted for at least 12 months, with LLPCs, RBD‐specific memory B cells, and respiratory T_RM_ cells detectable at 12 months. Vaccination protected adult mice and markedly reduced pulmonary viral loads in aged mice. (D) In rhesus macaques, NanoCF501/SSM2 induced RBD‐specific mucosal and systemic antibodies, neutralizing activity and sustained RBD‐specific T‐cell responses. LLPC, long‐lived plasma cell; T_RM_, tissue‐resident memory T.

Looking ahead, further research in several areas could facilitate the clinical translation of mucosal adjuvant–based vaccines. First, studies in large‐animal models, particularly non‐human primates, will be important for evaluating the durability of protective immunity and further elucidating the mechanisms underlying long‐term mucosal immune responses. Second, comparative evaluation of intranasal administration and aerosol inhalation could help identify delivery approaches suitable for human use and guide the development of appropriate delivery devices. Third, optimized formulations that promote the co‐delivery of antigen and adjuvant may enhance their coordinated activity and improve the consistency and efficacy of mucosal immune responses.

Overall, NanoCF501 combines a novel small‐molecule STING agonist with a nanoparticle‐based formulation strategy, enabling robust immune activation in the respiratory tract while limiting systemic exposure. These features support its potential as a mucosal adjuvant for respiratory vaccination and provide a basis for extending this strategy to other respiratory vaccine antigens.

## AUTHOR CONTRIBUTIONS

Weijie Wang drafted the manuscript. Muzhuo Wang contributed to the literature preparation. Zezhong Liu, Lu Lu and Shibo Jiang conceived the study idea and modified the draft.

## CONFLICT OF INTEREST STATEMENT

The authors declare no conflicts of interest.

## Data Availability

All data generated or analysed during this study are included in this published article.

## References

[1] Zhou X , Wu Y , Zhu Z , et al. Mucosal immune response in biology, disease prevention and treatment. Signal Transduct Target Ther. 2025;10(1):7. doi:10.1038/s41392‐024‐02043‐4 39774607 10.1038/s41392-024-02043-4 PMC11707400

[2] Kwon DI , Mao T , Israelow B , Santos Guedes de Sá K , Dong H , Iwasaki A . Mucosal unadjuvanted booster vaccines elicit local IgA responses by conversion of pre‐existing immunity in mice. Nat Immunol. 2025;26(6):908‐919. doi:10.1038/s41590‐025‐02156‐0 40360777 10.1038/s41590-025-02156-0 PMC12133566

[3] Jeyanathan M , Afkhami S , D'Agostino MR , et al. Induction of lung mucosal immunity by a next‐generation inhaled aerosol COVID‐19 vaccine: an open‐label, multi‐arm phase 1 clinical trial. Nat Commun. 2025;16(1):6000. doi:10.1038/s41467‐025‐60726‐0 40603330 10.1038/s41467-025-60726-0 PMC12223143

[4] McMahan K , Wegmann F , Aid M , et al. Mucosal boosting enhances vaccine protection against SARS‐CoV‐2 in macaques. Nature. 2024;626(7998):385‐391. doi:10.1038/s41586‐023‐06951‐3 38096903 10.1038/s41586-023-06951-3 PMC10849944

[5] Li W , Wang T , Rajendrakumar AM , et al. An FcRn‐targeted mucosal vaccine against SARS‐CoV‐2 infection and transmission. Nat Commun. 2023;14(1):7114. doi:10.1038/s41467‐023‐42796‐0 37932271 10.1038/s41467-023-42796-0 PMC10628175

[6] Wang S , Chen S , Dai L , et al. Regional immunity in the respiratory tract: challenges and opportunities. Sci Bull. 2026;71(11):2874‐2890. doi:10.1016/j.scib.2026.04.057 10.1016/j.scib.2026.04.057 42150938

[7] Hong W , Alu A , Zhang Z , et al. Mucosal immunity and vaccine development. Signal Transduct Target Ther. 2026;11(1):301. doi:10.1038/s41392‐026‐02687‐4 42503519 10.1038/s41392-026-02687-4 PMC13402638

[8] Zhao T , Cai Y , Jiang Y , et al. Vaccine adjuvants: mechanisms and platforms. Signal Transduct Target Ther. 2023;8(1):283. doi:10.1038/s41392‐023‐01557‐7 37468460 10.1038/s41392-023-01557-7 PMC10356842

[9] Ben‐Akiva E , Chapman A , Mao T , Irvine DJ . Linking vaccine adjuvant mechanisms of action to function. Sci Immunol. 2025;10(104):eado5937. doi:10.1126/sciimmunol.ado5937 39951545 10.1126/sciimmunol.ado5937 PMC13292272

[10] Lavelle EC , Ward RW . Mucosal vaccines—fortifying the frontiers. Nat Rev Immunol. 2022;22(4):236‐250. doi:10.1038/s41577‐021‐00583‐2 34312520 10.1038/s41577-021-00583-2 PMC8312369

[11] Liu Z , Chan JF , Zhou J , et al. A pan‐sarbecovirus vaccine induces highly potent and durable neutralizing antibody responses in non‐human primates against SARS‐CoV‐2 Omicron variant. Cell Res. 2022;32(5):495‐497. doi:10.1038/s41422‐022‐00631‐z 35210607 10.1038/s41422-022-00631-z PMC8866927

[12] Liu Z , Zhou J , Xu W , et al. A novel STING agonist‐adjuvanted pan‐sarbecovirus vaccine elicits potent and durable neutralizing antibody and T cell responses in mice, rabbits and NHPs. Cell Res. 2022;32(3):269‐287. doi:10.1038/s41422‐022‐00612‐2 35046518 10.1038/s41422-022-00612-2 PMC8767042

[13] Liu Z , Zhou J , Wang W , et al. Neutralization of SARS‐CoV‐2 BA.2.86 and JN.1 by CF501 adjuvant‐enhanced immune responses targeting the conserved epitopes in ancestral RBD. Cell Rep Med. 2024;5(3):101445. doi:10.1016/j.xcrm.2024.101445 38428429 10.1016/j.xcrm.2024.101445 PMC10983032

[14] Liu Z , Zhou J , Wang X , et al. A pan‐sarbecovirus vaccine based on RBD of SARS‐CoV‐2 original strain elicits potent neutralizing antibodies against XBB in non‐human primates. Proc Natl Acad Sci U S A. 2023;120(11):e2221713120. doi:10.1073/pnas.2221713120 36897979 10.1073/pnas.2221713120 PMC10242718

[15] Liu Z , Zhou J , Gao R , et al. Precision‐engineered STING agonist nanoparticles enable coordinated mucosal‐systemic immunity for durable pan‐β‐coronavirus protection. Nat Nanotechnol. 2026;21(7):1008‐1021. doi:10.1038/s41565‐026‐02188‐z 42310425 10.1038/s41565-026-02188-z

[16] Wang M , Wang W , Xu W , et al. Intranasal gold nanoparticle‐antigen induces mucosal and systemic immunity against β‐coronaviruses. hLife. 2026. doi:10.1016/j.hlife.2026.07.002

